# Immunoscore and Immunoprofiling in cancer: an update from the melanoma and immunotherapy bridge 2015

**DOI:** 10.1186/s12967-016-1029-z

**Published:** 2016-09-20

**Authors:** J. Galon, B. A. Fox, C. B. Bifulco, G. Masucci, T. Rau, G. Botti, F. M. Marincola, G. Ciliberto, F. Pages, P. A. Ascierto, M. Capone

**Affiliations:** 1 Laboratory of Integrative Cancer Immunology, INSERM UMRS1138 Cordeliers Research Center, University Pierre et Marie Curie, Paris 6, 15 Rue de l’Ecole de Medecine, 75006 Paris, France; 2 University Paris Descartes, 45 Rue Saints Pères, 75006 Paris, France; 3 Robert W. Franz Cancer Research Center, Earle A. Chiles Research Institute, Providence Cancer Center, Providence Portland Medical Center, Portland, OR 97213 USA; 4 Department of Molecular Microbiology & Immunology, Oregon Health & Science University, Portland, OR 97239 USA; 5 Department of Pathology, Providence Portland Medical Center, Portland, OR 97213 USA; 6 Department of Oncology-Pathology, The Karolinska Hospital, Stockholm, Sweden; 7 Institute of Pathology, University of Bern, Bern, Switzerland; 8 Unit of Pathology, IRCCS, Istituto Nazionale Tumori, Fondazione “G. Pascale”, Naples, Italy; 9 Sidra Medical and Research Center, Doha, Qatar; 10 IRCCS, Istituto Nazionale Tumori, Fondazione “G. Pascale”,Scentific Directorate, Naples, Italy; 11 Laboratory of Integrative Cancer Immunology, INSERM UMRS1138, Cordeliers Research Center, 15 Rue de l’Ecole de Medecine, 75006 Paris, France; 12 Centre de Recherche des Cordeliers, University Pierre et Marie Curie, Paris 6, 15 Rue de l’Ecole de Medecine, 75006 Paris, France; 13 Immunomonitoring Platform, Laboratory of Immunology, Georges Pompidou European Hospital, 20-40 Rue Leblanc, 75015 Paris, France; 14 Unit of Melanoma, Cancer Immunotherapy and Innovative Therapy, Istituto Nazionale Tumori, Fondazione “G. Pascale”, Naples, Italy

**Keywords:** Melanoma, Immunoscore, Immunoprofiling

## Abstract

The fifth “Melanoma Bridge Meeting” took place in Naples, December 1–5th, 2015. The main topics discussed at this meeting were: Molecular and Immuno advances, Immunotherapies and Combination Therapies, Tumor Microenvironment and Biomarkers and Immunoscore. The natural history of cancer involves interactions between the tumor and the immune system of the host. The immune infiltration at the tumor site may be indicative of host response. Significant correlations were shown between the levels of immune cell infiltration in tumors and patient’s clinical outcome. Moreover, incredible progress comes from the discovery of mutation-encoded tumor neoantigens. In fact, as tumors grow, they acquire mutations that are able to influence the response of patients to immune checkpoint inhibitors. It has been demonstrated that sensitivity to PD-1 and CTLA-4 blockade in patients with advanced NSCLC and melanoma was enhanced in tumors enriched for clonal neoantigens. The road ahead is still very long, but the knowledge of the mechanisms of immune escape, the study of tumor neo-antigens as well as of tumor microenvironment and the development of new immunotherapy strategies, will make cancer a more and more treatable disease.

## Background

Because cancer is a heterogeneous and dynamic microenvironment communicating with the immune system, the immune contexture of tumor microenvironment showed to be able influence the course of the disease. Hence, pre-existing immunity is determining the fate and survival of the patient and the likelihood of response to immunotherapy.

Quantification of immune cell densities (n = 415 patients, 6640 immunostains) revealed the major positive role of cytotoxic and memory T cells for patient’s survival [1]. These findings became the foundation of a new concept: immune contexture (i.e. nature, functional orientation, density, and location within distinct tumor regions, of a natural in situ immune reaction) might be used by immunoscore. Recently, many reports suggest that cancer development is controlled by the host’s immune system underlying the importance of including immunological biomarkers for the prediction of prognosis and response to therapy. For this reason, the impact of the immunoscore needs to be evaluated more thoroughly in other tumor types given the universal importance of the immune system in cancers.

## Update on Immunoscore Worldwide Consortium (SITC) and Immunoscore on other cancer types

### Immunoscore in colorectal cancer

The Immunoscore is now defined as a prognostic tool to use for quantification of in situ immune cell infiltrates, which appears to be superior to the tumor-node-metastasis (TNM) classification in colorectal cancer. In NSCLC, no Immunoscore has been established, but in situ tumor immunology is recognized as highly important [2]. The question still open is the challenge of the “universal” character of the Immunoscore.

In 2006 it was demonstrated that the immunological data (the type, density, and location of immune cells within the tumor samples) are a better predictor of patient survival than the histopathological methods currently used to stage colorectal cancer. An international consortium has been initiated to validate and promote the Immunoscore in routine clinical settings [3]. Specifically, an Immunoscore based on the combined analysis of CD8+ plus CD45RO+ cells in specific tumor regions was a useful criterion for the prediction of tumor recurrence and survival in patients with early-stage (AJCC/UICC-TNM stages I and II) CRC. Also, when considering the disease-specific survival according to CD8+ and CD45RO+ densities in combined tumor regions (central tumor/invasive margin, CT/IM), the scoring system allows to identify subgroups of patients with distinct clinical outcomes in terms of disease-free survival and overall survival [4]. The importance of the localized immune reaction in predicting recurrence and survival in patients with early-stage colorectal carcinoma has also been assessed [5].

The Immunoscore in colorectal cancer highlights the importance of digital scoring systems in surgical pathology. More specifically, in rectal cancers the evaluation of the impact of the immune infiltrate on prognosis in patients treated by primary surgery seemed to show significant differences between patient groups for survival times, with the poorest postoperative outcome in those with low densities of CD3 and CD8 in both central tumor and invasive margins [6]. It was also demonstrated that pre-existing CD8+ T cells distinctly located at the IM are associated with expression of the PD-1/PD-L1 immune inhibitory axis and may predict response to therapy [7]. In patients with refractory Hodgkin’s lymphoma treated with an anti PD-1 antibody (nivolumab) the objective response rate was 87 % [8].

The Immunoscore validation study aims at standardizing the procedures among centers through the provision of (a) recommendations for the IHC (CD3 & CD8) (b) a manual for the use of the software and (c) a tutorial to illustrate the drawing of the tumor regions in terms of heterogeneity (which clones of antibodies, staining automation and protocol, scanner used) and of non-heterogeneity (“Immunoscore module” of the software). The final results should be able to illustrate the robustness of the test.

Recently, Bern, Erlangen and Prague organized in a consortium now including data from more than 1000 CRC patients. Every site had to run through a digital workflow with tumor and stroma annotation, followed by detection of the invasive margin and automated quantification of the immune cells with CD3 and CD8 staining. The turn-around time of the whole digital analysis procedure has been estimated in 15 h, which seems reasonable also for clinical decision processes. Another point is how the digital analysis fits into the highly standardized pathology workflows existing in the clinics. The Immunoscore has the noteworthy advantage of being very close to routine pathology. The pathologist is engaged three times (blocking selection, marking the tumor area, that is time consuming and needs a special training, and reporting), still it is worth to integrate the Immunoscore owing to its capability to reflect the status of tumor infiltrating lymphocytes in the center and in the invasive margin. Patients were previously grouped by cut-offs for Kaplan Meyer curve calculation, losing valuable quantitative information. To overcome these two limitations, a more advanced software solution, which is faster, including an automatic detection of the tumor, and keeping the quantitative density values combined into a score, should be developed. It might be also overcome by reporting results in a scale format to enable clinicians to make a decision integrating the combination of markers and balancing the risks. This is feasible for the Immunoscore [9].

The Hôpital Européen Georges Pompidou of Paris, France (HEGP) developed an Immunomonitoring Platform in order to perform a quality control check at the initiation and at the end of the study. At the beginning of the study, adjacent slides were compared in the IHC in order to evaluate the quality and intensity of the staining. This permitted to create a tool to check the staining intensity. At the end of the study, all the immunostainings of each center were checked on the selection of the tumor region of interest, on the quality and intensities of the staining and on the detection of the stained cells.

As a prognostic tool in other cancers, high densities of CD3+ T cells, CD8+ cytotoxic T cells and CD45RO+ memory T cells are associated with a longer disease-free survival (after surgical resection of the primary tumor) and/or overall survival [10].

The Worldwide Immunoscore Consortium is systematically studying the Immunoscore as a new possible approach for the classification of cancer with the involvement of 23 centers, 17 Countries and more than 3000 patients. Three cohorts of colon cancer patients consisting into a training set, an internal validation set and an external validation set will be analyzed with a pre-defined analysis work plan by an external statistician, independent of the consortium. In other studies, the impact of Immunoscore on several types of cancer is being evaluated. A quantitative analysis of tumor-infiltrating lymphocytes (TILs) subsets within brain metastasis, coming from multiple primary tumors (mainly melanoma, breast, kidney, lung cancer), was performed and the prognostic impact of Immunoscore was evaluated. Immunoscore showed significant correlation with survival prognosis [11]. Furthermore, more prospective studies implementing the Immunoscore (as the one reported by Anitei et al.) [6] are yet needed in order to convert it from a prognostic tool into a predictive one.

### Immunoscore in melanoma

Immune-regulated pathways influence multiple aspects of tumor development and present multiple opportunities to gage its response in order to make treatment decisions regarding: (1) prognosis (the presence of the right immune effector cells is correlated with better prognosis and survival) (2) immunotherapy (manipulate the patient’s own immune system to respond to the tumor) and (3) chemotherapy (response may also be immune-related).

The definition of Immunoscore in melanoma, based on the complex intratumoral immune reaction, is becoming a difficult challenge. So far the value of the Immunoscore has been well established in patients with early-stage (stage I–II) colorectal cancer where the immune profile seems have higher prognostic importance than clinical features such as TNM-staging system [5].

The Immunoscore is evaluated in metastatic lymph node tissue because they represent an extremely interesting model for a number of reasons as it has jet been demonstrated: for adjuvant therapy (melanoma stage III), they are more accessible than visceral metastases and the risk for distant metastases is high, and at last [12–14]. Patients with a stage III disease can benefit from an adjuvant therapy (interferon, immunotherapy), in fact, the evaluation of the microenvironment in the lymph nodes could be important for patient selection, and In many cases, the metastatic lymph nodes from lymphectomy are the only available tissue.

On the other hand, concerns are raised about the Immunoscore in lymph nodes because they are constitutively rich in CD3 and CD20 lymphocytes and, also, the evaluation of the periphery of the tumor is particularly complex. Moreover, lymph node metastases may be different in terms of immune infiltration compared to other metastatic lesions. Clinical outcome and prognostic factors of superficial and deep lymph node dissection for stage III cutaneous melanoma were retrospectively studied in patients who underwent surgical lymph node dissection for metastases at the National Cancer Institute, Naples. One of the aims was to develop an algorithm for the evaluation of the different markers [15]. In the cell counts for each patient, no apparent differences were found in expression levels in the reactive lymph nodes between relapse and no relapse groups, except for CD8, in which there were more patients with high expressing cells in the relapse group. There were significant differences in the peri/intra ratio for both CD3 and CD8, with the ratio being higher in no relapse patients compared to relapse patients for both proteins. Similar differences were seen in FoxP3 and CD20. These preliminary data were used to develop a risk score currently investigated in a larger melanoma cohort. The algorithm was developed using the whole slide images from serial sections stained with different markers that were automatically identified from tumor marker slide. Region labels were then transferred to marker slides via image registration and cells were automatically counted for each region. The report of cell counts and region labels for each patient was analyzed in the onco-immune report [16].

In order to investigate the predictive power of Immunoscore, the MISIPI study has involved 200 FPFE samples from metastatic melanoma patients treated with Ipilimumab where density of different immune populations was assessed, using a digital image analysis application to characterize immune infiltrate expression of CD3, CD8, CD20, FoxP3 and CD163 and of PD-L1. The aim was to correlate marker expression profile with clinical outcome [17]. No relationship between CD3, CD8, CD20, CD163, FoxP3 both intratumoral (CT) and peritumoral (IM) with response/benefit was evidenced, but only a trend for the CD163 positive PD-L1 positive (CT) population (p = 0.07). CD8+ PD-L1− and higher and lower than median at IM seems to correlate with OS (p = 0.04). A similar correlation was found for CD163+ PD-L1+ (p = 0.05). The conjugated analysis presented even better correlation with overall survival (OS) (p = 0.01).

In the same time, a proof-of-concept study was designed with 31 patients (11 CR, 1 PR, 4 SD and 15 PD) on first line treatment with cisplatin + temodal or dacarbazine or other. Tissue sections were stained with H&E, CD3 and FoxP3. Co-expression analysis was performed on the basis of an automated alignment of serial sections in order to correlate cell density patterns with Ipilimumab patient OS. First results indicate that melanocyte-FoxP3 spatial relations are the most predictive factors and that, while CD3 alone does not provide substantial value, CD3/FoxP3 ratio on IM seems to be a promising additional factor.

## Immunoprofiling (next generation)

The success of immunotherapy for the treatment of metastatic melanoma is contingent on the identification of appropriate target antigens. As immunotherapy strategies become increasingly sophisticated and powerful, finding new biomarkers whose expression is predictive of the efficacy of immunotherapy is a major challenge to maximize patient tremendous growth in the understanding of the immune response to cancer is beginning to generate breakthroughs in cancer treatment. However, our understanding of immuno-oncology is far from complete. There is a need to explore the complex relationship between the immune system and primary tumors, in both the tumor microenvironment and in peripheral blood, as well as to robustly monitor changes in the immune response associated with potential therapeutic approaches.

It has been demonstrated that PD1–PDL1 blockade efficacy requires pre-existing adaptive immune resistance [7, 18]. In order to objectively determine PD-L1 protein levels, FDA approved 4 different companion diagnostic immunohistochemistry (IHC) tests for PD-L1 in NSCLC. However, both heterogeneity within tumors and prominent inter-assay variability or discordance has been found [19]. Two programs were started in order to improve the understanding of PD-L1 protein expression in lung cancer: “Rx/Dx Industry PD-L1 Blueprint Proposal” of FDA-AACR-ASCO and the “Multi-Institutional Analysis of Programmed Cell Death-Ligand 1 (PD-L1) Expression in Lung Cancer” of NCCN. First of all, a deep understanding is needed of the differences between innate (with constitutive oncogenic signaling inducing PD-L1 expression on tumor cells) and adaptive immune resistance (with T cell-induced PD-L1 upregulation) and of the interaction distance measurements. Multispectral fluorescent IHC with a 6-plex panel was used to analyze the tumor microenvironment in patients with melanoma to predict successful TIL generation. CD8 to FoxP3 ratio resulted to be predictive of ability to culture autologous TILs [2]. Using the same assay, Immuneprofiling was found to correlate to overall survival in NSCLC treated with conventional therapies. Finally, a head and neck squamous cell carcinoma (HNSCC) patients cohort was analyzed. Since most of HNSCC samples are primary tumor the invasive margin was tested first. Results indicate that both CD8+ T cells infiltrate in the tumor component of the invasive margin and PD-L1 expression in the tumor predict disease recurrence. Before the routine clinical application, a few steps will need to be covered: (a) the automation of the staining procedure (b) the “whole” digital multiplexed slides and consistent integration with H&E based workflows and (c) the development and validation of orthogonal approaches (e.g. Nanostring, RNAseq or Mass Spectrometry).

In the Stockholm cohort, it has been explored the prognostic impact of the Immune profile in colon cancer patients Dukes B and C, randomized to surgery or surgery and adjuvant treatment. When comparing surgery alone versus surgery and adjuvant treatment, the difference between Dukes B and C is significant, with better survival with surgery and adjuvant for Dukes B. Treatment does not seem to change the outcome. Four biomarkers were tested in the cohort: HLA-A*02, major histocompatibility complex (MHC) class I, HLA-G and CD8+ lymphocytes. HLA-A*02 is a common allele in the Scandinavian population, known as a negative prognostic factor and more important than the expression of MHC class I for patients with epithelial ovarian cancer. Duke C patients with HLA-A*02 genotype randomized to surgery only had a worst outcome, but it was not the case for patients with other HLA-A genotype. Also, women with HLA-A*02 genotype with Dukes C treated by surgery had worst prognosis but could improve the prognosis if treated with adjuvant chemotherapy. MHC class I showed a paradox, with better survival in the “absent” MHC subgroup. HLA-G protein expression on tumor cells was found to be a significant marker in men. Also CD8+ cytotoxic T cells at the tumor microenvironment was found to be a significant marker in men compared to women. Women seem to have a worst prognosis if they are HLA-A*02 independent of CD8+ infiltration status, while men seem to have a worst prognosis with low CD8+ infiltration independent of HLA-A genotype. Moreover, CD8+ infiltration resulted to be predictive for survival in the whole cohort and the absence of HLA-A*02 genotype to be predictive for good survival in the female subgroup compared to CD8+ .

## Discussion and conclusion

Despite the steady progress and the evidence about the importance of Immunoscore, several questions remain open. The first unanswered questions are how to explain “hot” and “cold” immune infiltrated tumors and what are the mechanisms associated with TIL infiltration. A number of contributing systems have been proposed, as mutations driver, chromosomal instability, T cell proliferation [20] or T-cell attraction [21, 22]. When examining the chromosomal instability, mutation patterns, and gene expression profiling in 270 microsatellite instability-high (MSI-H) and in microsatellite stable (MSS) patients, those with microsatellite instability-high (MSI-H) showed multiple Frameshift mutations. Also, major histocompatibility complex (MHC) Class I and II seem to have predictable immunogenic frameshift mutations on different HLA molecules. The genetic analysis of missense and frameshift immunogenic mutations (epitopes) compared to nonsense (silent) mutations seem to indicate the genetic evidence of immunoediting, owing to a decrement in frameshift immunogenic mutations in MSI patients compared to silent mutations and to less than expected number of missense immunogenic mutations in CRC patients, and particularly MSI patients compared to silent mutations. On the other hand, Immunoscore high patients have prolonged survival regardless of the MSI status [23].

Numerous analyses of large patient cohorts identified specific patterns of immune activation associated with patient survival. We established these as the immune contexture, encompassing the type, functional orientation, density and location of adaptive immune cells within distinct tumor regions. Based on the immune contexture, a standardized, powerful immune stratification system, the Immunoscore, was delineated. The immune contexture is characterized by immune signatures also observed in association with the broader phenomenon of immune-mediated, tissue-specific destruction. We defined these as the immunologic constant of rejection. Predictive, prognostic, and mechanistic immune signatures overlap, and a continuum of intratumoral immune reactions exists. The balance between tumor cell growth and elimination may be tipped upon a crescendo induced by immune manipulations aimed at enhancing naturally occurring immunosurveillance. We propose a broader immunological interpretation of these three concepts—immune contexture, Immunoscore, and immunologic constant of rejection—that segregates oncogenic processes independently of their tissue origin. The immune contexture is defined as the type, functional orientation, density, and location of adaptive immune cells within distinct tumor regions. The Immunoscore is derived from three aspects of the immune contexture: the type, density, and location of immune cells. The functional orientation of the immune contexture is characterized by immune signatures qualitatively similar to those predicting response to immunotherapy, which are observable in association with the broader phenomenon of immune-mediated, tissue-specific destruction. These signatures are detectable during regression of cancer following immunotherapy, allograft rejection, Graft versus Host Disease, flares of autoimmunity, or destruction of virally infected cells to clear intracellular pathogens. We defined them as the immunologic constant of rejection. Intratumoral immune biomarkers measure the status of activation of a naturally protective mechanism, which, if successfully elicited, will lead to tumor destruction. Thus, with a few exceptions, it is probable that predictive immune biomarkers will overlap with the prognostic and the mechanistic ones. Although the redundancy between prognostic, predictive, and mechanistic immune signatures could seem obvious, the basis of its molecular continuum has only been recently proposed [24].

To well define Immunoscore in melanoma, it needs to evaluate additional cohort with the Immunoscore-in-lymph-nodes algorithm and to verify the possibility of scoring the immuno-infiltrate in melanoma also analyzing the functionality of the different immune cells. It will be important to evaluate the immuno-infiltrate in lymph nodes in a large cohort of patients and to correlate this with the outcome from adjuvant treatments, as well as to go further with the Immunoprofiling analysis (IDO, TIM-3, CD137, etc.)

In conclusion, the next steps to promote the Immunoscore in routine clinical settings include the reinforcement of the demonstration of its prognostic value, of its predictive value and the identification of the follow-up parameters that could modify the initial prognostic power of the Immunoscore.

## Abbreviations

TNM: tumor-node-metastasis
CT: central tumor
IM: invasive margin
TILs: tumor-infiltrating lymphocytes
OS: overall survival
MSI-H: microsatellite instability-high
IHC: immunohistochemistry
